# Unmet need for mental health care among adolescents in Asia and Europe

**DOI:** 10.1007/s00787-024-02472-0

**Published:** 2024-05-31

**Authors:** Yuko Mori, Andre Sourander, Kaisa Mishina, Tiia Ståhlberg, Anat Brunstein Klomek, Gerasimos Kolaitis, Hitoshi Kaneko, Liping Li, Mai Nguyen Huong, Samir Kumar Praharaj, Henriette Kyrrestad, Lotta Lempinen, Emmi Heinonen

**Affiliations:** 1 Department of Child Psychiatry, Research Centre for Child Psychiatry, University of Turku and Turku University Hospital, Lemminkäisenkatu 3, Turku, 20014 Finland; 2 INVEST Research Flagship Center, University of Turku, Turku, Finland; 3 Department of Child Psychiatry, Turku University Hospital, Turku, Finland; 4 Baruch Ivcher School of Psychology, Reichman University, Herzliya, Israel; 5 Department of Child Psychiatry, School of Medicine, National and Kapodistrian University of Athens, Aghia Sophia Children’s Hospital, Athens, Greece; 6 Psychological Support and Research Center for Human Development, Nagoya University, Nagoya, Japan; 7 School of Public Health, Shantou University Medical College, Shantou, China; 8 Department of Psychiatry, Vietnam National Children’s Hospital, Hanoi, Vietnam; 9 Department of Psychiatry, Kasturba Medical College, Manipal, India; 10 Manipal Academy of Higher Education, Manipal, India; 11 Regional Centre for Child and Youth Mental Health and Child Welfare, Faculty of Health Sciences, UiT The Arctic University of Norway, Tromsø, Norway

**Keywords:** Unmet need, Help-seeking, Mental health, Adolescent, Cross-cultural

## Abstract

The unmet need for mental health care is a global concern. There is a lack of cross-cultural studies examining adolescent help-seeking behavior from both formal and informal sources, including both high-and lower-income countries. This study investigates mental health help-seeking behavior in eight Asian and European countries. Data from 13,184 adolescents aged 13–15 (51% girls) was analysed using mixed-effects logistic regression with school-wise random intercepts to compare countries and genders. Although a significant proportion of adolescents considered getting or sought informal help, formal help-seeking remained exceptionally low, especially in middle-income countries (< 1%), while it ranged from 2 to 7% in high-income countries. Among adolescents with high emotional and behavioral problems (scoring above the 90th percentile on the Strengths and Difficulties Questionnaire), 1–2% of those in middle-income countries and 6–25% of those in high-income countries sought formal help. Girls generally seek more help than boys. The study shows the most adolescents do not receive formal help for mental health problems. The unmet need gap is enormous, especially in lower-income countries. Informal sources of support, including relatives, peers, and teachers, play a crucial role, especially in lower-income countries.

**Supplementary Information:**

The online version contains supplementary material available at 10.1007/s00787-024-02472-0.

## Introduction

It is estimated that one in seven adolescents experience any diagnosed mental health disorder globally [1]. The peak age of onset for any mental disorder is 14.5 years, and the median age of onset is 18 years, indicating the critical importance of treatment during childhood and adolescence [2]. The referral process for children and adolescents to mental health services can involve multiple sources, including parents, medical professionals, and adolescents themselves. However, previous reviews have indicated low help-seeking behavior among adolescents even in developed nations with relatively accessible services [3]. A significant proportion of adolescents with mental health problems do not receive any mental health services [4]. This gap between the high prevalence of mental health disorders and low treatment utilization has been recognized as a matter of worldwide concern for policymakers, clinicians, and researchers [5, 6].

Mental health help-seeking behavior is an adaptive coping process that attempts to obtain external help to deal with mental health issues. It includes both informal (e.g., family, peers, and teachers) and formal (e.g., health or social sector professionals) sources of help [7]. The help-seeking pathway generally starts with the recognition of problems followed by consideration of seeking help and the decision to seek help [8]. Adolescents commonly fail to seek help because of poor mental health literacy, stigma, embarrassment, and systematic barriers, such as costs and availability of services [3]. Adolescents are more likely to seek formal help if they are older, experience multiple issues, have positive previous experiences with services, and if their parents perceive burden caused by their problems [5, 9]. It has been estimated that more than half of adolescents have unmet need for mental health care globally [10]. The definition of unmet need for mental healthcare varies across studies. It can be considered as the absence of both informal and formal mental health care for people with mental health problems, but it traditionally and generally refers to the absence of formal care [10–12]. It has been shown in the adult population that the unmet need is higher in lower-income countries than in high-income countries [13].

There are several major limitations to the existing studies on help-seeking behavior. First, although nearly 90% of children and adolescents live in low- and middle-income countries [14], existing evidence is largely from a small number of high-income Western countries [10, 15]. Second, cross-national research on adolescent populations is clearly lacking [10]. Third, there is a lack of cross-national research on adolescent help-seeking behavior, including consideration of seeking help and seeking help from both informal and formal sources. Most studies on help-seeking behavior have examined only formal help-seeking behavior in Western countries [5, 10]. Informal sources are important to examine because such sources play a central role and are often serving as the first agents in the help-seeking process for young people with mental health problems [16]. Including the aspects of seeking help provides insights into the multistep process, facilitating a comprehensive understanding of help-seeking behavior [17]. This study examines both informal and formal help-seeking behavior using a large multinational sample of adolescents with widely differing income levels.

To the best of our knowledge, this is the first large cross-national study of both informal and formal help-seeking behavior for mental health problems among adolescents, including 16,845 adolescents aged 13–15 years from eight Asian and European high to middle-income level countries. The first aim was to investigate how formal and informal help-seeking behavior for mental health problems among adolescents differs by country and gender. The second aim was to examine the unmet need for formal mental health help in different countries.

## Methods

Data were collected as part of a large-scale cross-national study, the Eurasian Child Mental Health Study (EACMHS), a collaborative study on the well-being and mental health of children and adolescents using a standardized methodological approach across participating countries [18, 19]. The participating countries include 13 Asian and European countries with widely differing income levels based on the World Bank classification [20]. The countries included in the present study were eight Asian and European countries: Norway, Finland, Greece, Israel, Japan, India, Vietnam, and China. The selection of these eight countries was based on the availability of complete data on help-seeking behavior. All sites had ethical approval from the institutional review boards of each country, and the researchers obtained permission from the schools. Participation was voluntary, and the anonymity and confidentiality of the participants were ensured. The researchers obtained consent from the parents or school authorities according to each country’s policies at the time of the study. This study was performed in accordance with the ethical standards of the Declaration of Helsinki and its later amendments.

### Overview of adolescent mental health services across countries

The participating countries vary significantly in terms of their mental healthcare systems and available resources for adolescents’ mental health. Norway and Finland stand out for their accessible and free mental health services for adolescents, supported by school-based programs and a high ratio of mental health workers per population [21, 22]. Greece, Israel, and Japan also prioritize accessibility, with various community-based and school-focused initiatives [23–25]. While efforts are being made to ensure broader accessibility of mental health support, India, Vietnam and China face challenges with limited resources and regional disparities in accessibility [26–28]. For example, in a cross-national comparison from 2018, the ratio of child and adolescent psychiatrists per 100,000 children is significantly limited in China (0.09), India (0.02), and Vietnam (0.00) while Norway and Finland have the highest ratios, with 47.74 and 45.40 respectively, followed by Greece (22.24), Israel (11.29) and Japan (1.82) [18]. Mental health service in lower income countries remains largely inadequate and the lack of services is particularly acute in remote regions [29].

### Sample

For a description of the study sample, see Table 1. Participants in the EACMHS were drawn from 118 schools comprising 16,845 adolescents from eight countries. This survey was conducted between 2011 and 2017. The response rate ranged from 76.6% in Israel to 96.1% in China. The analysis was restricted to adolescents aged 13 to 15 years (*n* = 13,283) because most students were within this age range and to make the data more comparable. Ninety-nine of the 13,283 participating adolescents were excluded based on missing relevant data, and the final analysis included 13,184 adolescents with a mean age of 13.9 years (51.0% girls). The sample sizes ranged from 920 (Vietnam) to 2,946 (Finland).

**Table 1:** Description of the study sample by country

Country	Income classification	Survey year	Original total sample	Final sample size	Response rate	Girls	Age	Rural residence	Urban residence	Public school	Private school	Schools
			*n*	*n*	%	*n* (%)	Mean	*n* (%)	*n* (%)	*n* (%)	*n* (%)	*n*
Asia												
India	LM	2016	2016	1597	93.9	828 (51.9)	13.6	240 (15.0)	1357 (85.0)	198 (12.4)	1399 (87.6)	11
Vietnam	LM	2016	1118	920	93.2	471 (51.2)	13.9	0 (0.0)	920 (100)	920 (100)	0 (0.0)	3
China	UM	2016	2659	2043	96.1	1008 (49.3)	13.8	1250 (61.2)	793 (38.8)	1605 (78.6)	438 (21.4)	10
Japan	H	2011	1842	1789	92.8	924 (51.7)	13.9	971 (54.3)	818 (45.7)	1789 (100)	0 (0.0)	17
Europe												
Finland	H	2014	3422	2946	91.9	1478 (50.2)	14.1	293 (10.0)	2653 (90.0)	2946 (100)	0 (0.0)	13
Greece	H	2016	1581	1028	About 85	549 (53.4)^a^	13.6	283 (27.5)	745 (72.5)	1028 (100)	0 (0.0)	14
Israel	H	2014	2188	1023	76.6	553 (54.1)^a^	14.0	0 (0.0)	1023 (100)	1023 (100)	0 (0.0)	5
Norway	H	2017	2019	1838	n/a^b^	910 (49.5)	13.9	283 (15.4)	1555 (84.6)	1734 (99.4)	10 (0.6)	45
Total	LM-H	2011–2017	16,845	13,184	76.6–96.1	6,721 (51.0)	13.9	3320 (25.2)	9864 (74.8)	11,233 (85.9)	1847 (14.1)	118

The participating countries are listed by their income level (low to high) by Asian and European countries. The chi-square test for equal proportions was used to analyse gender distribution. H: high income; UM: upper-middle income; LM: lower-middle income. The income level classification was based on the World Bank classification for the year of the survey in each country. ^a^ There were significantly more girls than boys in Greece and Israel (*p* < 0.05). ^b^ Information on response rate is not available in Norway

### Questionnaire and procedure

The questionnaire for this cross-national study was developed based on a self-report survey used among adolescents in Finland [30, 31]. The questionnaires were translated into the local language. A back-translation process was employed to ensure translation accuracy. The questionnaire included the same measures (mandatory questions) and optional survey questions which countries can choose depending on the national importance of questions and ethics approval requirements. The survey was conducted within the classroom, and all students at school at the time of the survey were invited to participate during school hours. The questionnaires were administered by the teachers and returned to the researchers anonymously. Convenience sampling method was chosen due to substantial time and budget limitations. The aim was to select schools represent the diversity of the education system in each participating country, considering factors like urban/rural distribution and socioeconomic status. Data collection was performed in the same way in all countries to establish equivalence, except for the format of the questionnaire. The paper-format questionnaires were administered in seven countries, whereas in Norway, the questionnaire was administered electronically.

### Measures

Sociodemographic factors included age, gender, geographic location of the school, and type of school. Help-seeking behavior in the past six months was measured by two items. In the first question, the adolescents were asked the following question:“within the past six months, have you at any point felt a need for outside help (someone outside your immediate family) with your problems, feelings, behavior or emotional trouble?”. The possible answers were “no, I have not felt the need”, “I have considered getting outside help” and “I have sought outside help”. The second question was only for those who sought help. It asked about the sources of help used, and adolescents could choose more than one source, including informal sources such as relatives and teachers, formal sources such as school nurses, psychologists, and counselors, or ‘someone else’. The ‘someone else’ category was open-ended and two authors (YM and EH) categorized the responses into informal and formal help-seeking. Any disagreements were consulted by senior researchers (AS and KM) onsite; if needed, senior researchers in the participating countries were consulted.

Adolescents’ emotional and behavioral difficulties were measured using a self-report version of the Strengths and Difficulties Questionnaire (SDQ), comprising 25 items, along with one impact supplement question. The SDQ has five scales: emotional problems, conduct problems, hyperactivity, peer problems, and prosocial. The scales were combined (excluding the prosocial scale) into a total difficulties score (SDQ total scores). The SDQ is a reliable and validated tool used for standardized measures of youth psychopathology [32]. Internal consistency was acceptable (Table S1, available online). In the absence of normative data for all the study countries, the 90th percentile cut-off points were identified for each country sample based on the distribution of SDQ total scores in each sample following Goodman’s epidemiological study [32]. Previous studies have also shown that SDQ total scores above the 90th percentile are strongly associated with the need for formal help [33]. An impact supplement of the SDQ was used to identify adolescents with perceived difficulties [34]. The adolescents were asked if they had difficulties with their emotions, concentration, behavior, or getting along with others. The possible answers were no, yes-minor difficulties, yes-definite difficulties, and yes-severe difficulties.

### Data analysis

The responses from all countries were pooled to create a descriptive analysis. The proportion of adolescents who (i) perceived no need for help, (ii) considered getting help, (iii) received help from informal sources, and (iv) received help from formal sources was estimated. Adolescents who sought help from both informal and formal sources were classified into the formal source group. To compare how help-seeking differs between girls and boys in each country, mixed-effects multinomial logistic regression with school-wise random intercepts was performed, adjusting for age. Since the effect of gender was significant (*p* < 0.1) in all countries except India and significant interactions were found between gender and other explanatory variables in the total sample, further analysis was conducted separately for girls and boys. In the present study, unmet mental health need is defined as the absence of support from health professionals among those with high emotional and behavioural difficulties [10]. The unmet need for mental health care was examined by calculating the proportion of adolescents who did not receive formal help among those with above 90th percentile SDQ total scores for each country. The association between the SDQ total scores and perceived difficulties was examined to validate the use of the 90th percentile SDQ total scores. This was done in only six countries because the perceived difficulties item was missing from Japan and Israel. The difference between girls and boys on the unmet need was examined using a two-tailed Fisher’s exact test. To compare country variation, a mixed-effects logistic regression was performed with school-wise random intercepts with reference to the country with the lowest unmet need, adjusting for age. Statistical significance was considered at *p* < 0.05, except in the gender interaction analysis, where a p-value less than 0.1 was considered significant. Statistical analyses were conducted using the SAS software (version 9.4; SAS Institute Inc. Cary, NC, USA, 2012).

## Results

Table 2 shows the help-seeking behavior in each country by gender (see Table S2 for the total sample, available online). Because of the significant effects of gender and country on help-seeking behavior, the results are presented separately for girls and boys. Of the 13,184 adolescents included in the analyses, 19.2% of adolescents (girls, 21.9%; boys, 16.3%) considered getting help, 6.3% of adolescents (girls, 7.7%; boys, 4.8%) sought help from informal sources, and merely 3.3% of them (girls, 4.9%; boys, 1.7%) sought help from formal sources. The proportion of adolescents who considered getting help between countries ranged from 10.3 to 36.4% (girls, 15.5–38.5%; boys, 5.0–34.4%). In most countries, formal help-seeking was limited. Despite the high perceived need ranging from 28.3 to 48.5% (girls, 25.8–53.6%; boys, 30.9–43.6%), 0.8–0.9% (girls, 0.6–0.9%; boys, 0.9–1.2%) in middle-income countries (India, Vietnam, China) sought formal help. In high-income countries, Greece, Israel, and Japan, only 2.0–2.5% (girls, 2.2–3.1%; boys, 0.9–2.9%) sought formal help. The two exceptions were Norway and Finland, where 6.4% and 6.9% (girls, 10.1% and 11.5%; boys, 2.2% and 2.8%, respectively) sought formal help. Girls were significantly more likely to seek help than boys in most countries, while in no country boys had higher help-seeking behavior than girls.

**Table 2:** The past 6 months help-seeking among girls and boys by country

Country		Total. *N*	Considered getting help, %	Sought informal help, %	Sought formal help, %	Pooled^a^, %
Asia						
India	Girls	828	18.6	6.6	0.6	25.8
	Boys	769	23.5	6.2	1.2	30.9
Vietnam	Girls	471	25.1*	15.9*	0.9	41.8**
	Boys	449	20.5	11.6	0.9	33.0
China	Girls	1008	38.5***	14.5***	0.6	53.6***
	Boys	1035	34.4	8.2	1.0	43.6
Japan	Girls	924	17.6	10.1***	2.7**	30.4***
	Boys	865	16.1	5.4	1.2	22.7
Europe						
Finland	Girls	1478	15.5***	3.5***	11.5***	30.4***
	Boys	1468	5.0	1.6	2.2	8.8
Greece	Girls	549	23.1***	8.6	2.2	33.9***
	Boys	479	14.0	6.5	2.9	23.4
Israel	Girls	553	16.8	1.5	3.1*	21.3
	Boys	470	14.9	1.1	0.9	16.8
Norway	Girls	910	22.3***	4.4***	10.1***	36.8***
	Boys	928	7.9	2.3	2.8	12.9
Total	Girls	6721	21.9***	7.7***	4.9***	34.5***
	Boys	6463	16.3	4.8	1.7	22.8

The participating countries are listed by their income level (low to high) by Asian and European countries. ^a^ The pooled percentage of considered getting help, sought informal help, and sought formal help. The difference between girls and boys was examined by mixed multinomial logistic regression adjusted by age (*p < 0.05, **p < 0.01, ***p < 0.001)

Table 3shows the proportion of different sources of help used by adolescents who had sought help in each country by gender. In this descriptive analysis, informal sources of help was included, even if the adolescent had also sought a formal source of help. Adolescents reported various sources of help for the ‘someone else’ option, including siblings, significant others, and internet friends. The most common ‘someone else’ source was ‘friends’, while in Israel, there was no data available on ‘someone else’ option. Non-human sources of help such as pets and God were excluded. Among adolescents who sought help, approximately 90% sought informal help in all Asian countries, ranging from 91.7% (India) to 96.1% (China) for girls and 87.7% (India) to 95.8% (China) for boys. In India, Vietnam, and China, more girls sought help from friends than boys, and help from relatives were more common among boys than girls in China and Japan. In India, more boys sought help from teachers than girls. On the other hand, formal help in Asian countries ranged from 3.9% (China) to 21.2,% (Japan) for girls and 7.1% (Vietnam) to 17.5% (Japan) for boys. A significant gender difference was found only in China with more boys seeking help from school nurses and medical doctors than girls. Formal sources of help were used more frequently in European countries, especially Finland, Israel, and Norway. In Greece, 81.4% of girls and 75.6% of boys sought informal help, whereas formal assistance was sought by 20.3% of girls and 31.1% of boys. Adolescents sought help from both informal and formal sources, but more from formal sources of help in Finland (informal, 42.5% for girls and 57.1% of boys; formal, 76.9% of girls and 57.1% of boys), Israel (informal, 52.0% of girls and 66.7% of boys; formal, 68.0% of girls and 44.4% of boys), and Norway (informal, 48.5% of girls and 63.8% of boys; formal, 69.7% of girls and 55.3% of boys). A significant gender difference was found in Finland with more girls seeking help from formal source of help, especially from psychologists and school counsellors, than boys. Moreover, seeking help form school nurses were more common among girls than boys in Norway.

**Table 3:** The sources of help that adolescents used by gender in each country among those who sought help

		Informal source of help, %					Formal source of help, %				
**Country** (***n*** = **number of adolescents who sought help)**		**Informal total**	**Friends**	**Relative**	**Teacher**	**Others**	**Formal total**	**Psychologist / school counsellor**	**School nurse**	**Medical doctor**	**Others**
Asia											
India	Girls (*n* = 60)	91.7	53.3**	30.0	11.7**	1.7	8.3	3.3	1.7	5.0	0.0
	Boys (*n* = 57)	87.7	24.6	33.3	36.8	0.0	15.8	5.3	0.0	10.5	0.0
Vietnam	Girls (*n* = 79)	94.9	45.6**	26.6	26.6	1.3	5.1	0.0	2.5	2.5	0.0
	Boys (*n* = 56)	92.9	23.2	30.4	35.7	5.4	7.1	0.0	3.6	3.6	0.0
China	Girls (*n* = 152)	96.1	40.8**	30.3*	21.1	12.5	3.9	3.9	0.0*	0.7**	0.0
	Boys (*n* = 95)	95.8	25.3	43.2	28.4	8.4	10.5	6.3	4.2	7.4	0.0
Japan	Girls (*n* = 118)	92.4	16.9	19.5**	63.6	6.7	21.2	4.2^a^	16.9^b^	2.5	0.8
	Boys (*n* = 57)	94.7	12.3	40.4	50.9	7.1	17.5	7.0^a^	10.5^b^	1.8	1.8
Europe											
Finland	Girls (*n* = 221)	42.5	19.9	19.5	8.6	3.2	76.9**	57.9**	33.0	12.7	14.5
	Boys (*n* = 56)	57.1	25.0	21.4	8.9	7.2	57.1	33.9	19.6	19.6	7.1
Greece	Girls (*n* = 59)	81.4	55.9	16.9	11.9	1.7	20.3	20.3	0.0	0.0	0.0
	Boys (*n* = 45)	75.6	48.9	28.9	6.7	0.0	31.1	24.4	2.2	4.4	0.0
Israel	Girls (*n* = 25)	52.0	NA	48.0	12.0	NA	68.0	60.0	0.0^c^	8.0	NA
	Boys (*n* = 9)	66.7	NA	44.4	22.2	NA	44.4	44.4	0.0^c^	0.0	NA
Norway	Girls (*n* = 132)	48.5	23.5	15.9	15.9	4.6	69.7	42.4	37.1**	16.7	6.8
	Boys (*n* = 47)	63.8	21.3	23.4	19.1	10.6	55.3	34.0	12.8	8.5	6.4

The participating countries are listed by their income level (low to high) by Asian and European countries. The total percentage of informal and formal sources of help exceeded 100% in some countries because the question about the source of help was in multiple-choice form. Israel did not have available data on ‘someone else’ option

^a^ Only school counsellor, ^b^ Nursing teacher, ^c^ In Israel, there are no school nurses who are always present at school. The difference between girls and boys was examined by Fisher’s exact test (*p < 0.05, **p < 0.01, ***p < 0.001)

Table 4 shows the proportion of adolescents with a high level of emotional and behavioral problems based on the SDQ total scores above the 90th percentile. A total of 11.1% of adolescents (girls, 13.2%; boys, 9.0%) had a high level of problems, that is, above the 90th percentile SDQ total scores (see Table S3 for the total sample, available online). In middle-income countries (India, Vietnam, China), 1–2% (girls, 1.8–2.3%; boys, 0.0–2.5%) of those with a high level of problems sought formal help in the past six months. In high-income countries, including Greece, Israel, and Japan, 5.5–7.1% (girls, 5.9–9.2%; boys, 2.6–8.9%) of those with a high level of problems sought formal help. In the two Nordic welfare countries, Finland and Norway, formal help-seeking was higher: 25.4% (girls, 32.8%; boys, 9.1%) and 20.6% (girls, 26.4%; boys, 10.3%) respectively of those with a high level of problems. Among boys, formal help-seeking was at the same level as in other high-income countries (9.1% and 10.3%, respectively). Girls had exceptionally high level of formal help-seeking and low unmet need in these two Nordic countries (32.8% and 26.4% respectively). Although help-seeking was more common among girls than boys in general, the significant difference was found only in Finland and Norway.

**Table 4:** The use of formal help among adolescents scoring above 90th percentile total difficulties scores

Country	Total *n*	Above 90th percentile SDQ total scores *n* (%)	Formal help/adolescents with above 90th percentile SDQ total scores^a^ *n* (%)	OR for unmet need (95% CI)
Girls				
Finland	1476	244 (16.5)	80 (32.8)	1
Norway	906	140 (15.5)	37 (26.4)	1.38 (0.86–2.23)
Greece	549	68 (12.4)	4 (5.9)	8.03*** (2.74–23.53)
Israel	543	65 (12.0)	6 (9.2)	4.90*** (1.98–12.15)
Japan	922	118 (12.8)	8 (6.8)	6.60*** (2.93–14.83)
India	811	88 (10.9)	2 (2.3)	21.87*** (5.09–93.87)
Vietnam	471	46 (9.8)	1 (2.2)	22.65** (2.97–172.75)
China	1006	113 (11.2)	2 (1.8)	28.49*** (6.67–121.61)
Total	6684	882 (13.2)	140 (15.9)	
Boys				
Finland	1465	110 (7.5)	10 (9.1)	1
Norway	925	78 (8.4)	8 (10.3)	0.68 (0.25–1.90)
Greece	475	45 (9.5)	4 (8.9)	0.77 (0.21–2.85)
Israel	457	39 (8.5)	1 (2.6)	3.48 (0.42–29.16)
Japan	861	82 (9.5)	3 (3.7)	2.44 (0.56–10.64)
India	761	81 (10.6)	2 (2.5)	3.61 (0.74–17.56)
Vietnam	449	47 (10.5)	0 (0.0)	-
China	1033	94 (9.1)	0 (0.0)	-
Total	6426	576 (9.0)	28 (4.9)	

SDQ: Strength and Difficulties Questionnaire. OR: odds ratio. ^a^ The percentage of those who received formal help out of those who were above 90th percentile of total difficulties score. *p < 0.05, **p < 0.01, ***p < 0.001

**Fig. 1:**
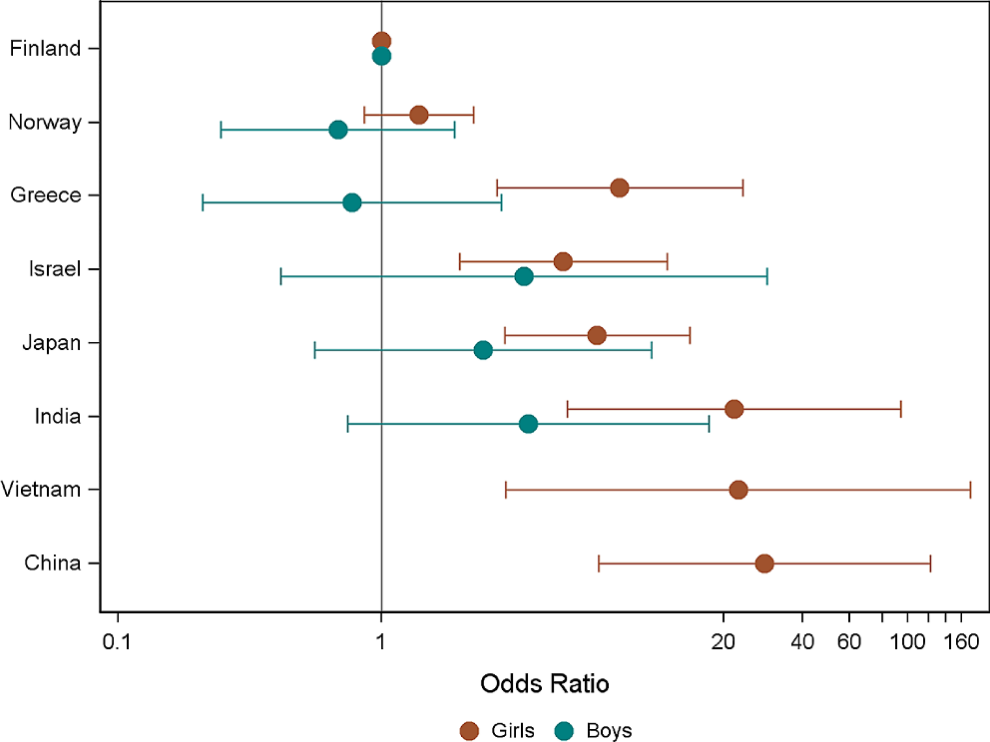
Odds ratios and 95% confidence intervals for not seeking formal help among those scoring above 90th percentile total difficulties scores

Figure 1 illustrates the differences in unmet need between countries. The odds of not seeking formal help among those with a high level of emotional and behavioral problems was estimated in the different countries, compared to the reference country with the lowest unmet need (Finland). All countries had increased odds of unmet need, except in Norway for girls. The greatest odds were in China (OR 28.49, 95% CI 6.67 − 121.61). For boys, there was no significant difference between countries and the odds ranged from 0.68 (95% CI 0.25–1.90) in Norway to 3.61 (95% CI 0.74–17.56) in India. There were no OR estimates for boys in China and Vietnam because none of the boys in these countries with a high level of problems had sought any formal help.

In the additional analysis (Table S4, available online), the associations of those who reported a high level of emotional and behavioral problems with self-report of perceived difficulties measured by the impact supplement question of SDQ were examined. Among those scoring above the 90th percentile on SDQ total scores, 70.9% (India) to 93.5% (Vietnam) of girls and 65.8% (India) to 89.4% (Vietnam) of boys had at least mild perceived difficulties.

## Discussion

Despite the high prevalence and significant associated burden, there is an enormous gap worldwide in the provision of professional care for mental disorders in adolescents. In all countries, the rate of considering or seeking help for mental health problems was considerably high. In the total sample, nearly one in three adolescents considered getting help or have sought help. However, the rate of seeking formal help was extremely low (< 1%) in all middle-income countries while in high-income countries, it ranged from 3 to 12% for girls to 1–3% for boys. The rate of seeking formal help (2–3%) among boys in the two Nordic countries, Norway and Finland, with well-developed public health services did not differ from other high-income countries while girls in these two Nordic countries had an exceptionally high rate of seeking formal help (10–12%).

In middle-income countries (India, Vietnam, China), very few adolescents (1–2%) with a high level of emotional and behavioural problems sought formal help. Since the study included China and India, the two countries with the highest populations of children and adolescents, this indicates that 98% or more of adolescents with self-reported high level of problems in most parts of the world may not have any access to formal help. The rate of seeking professional help was considerably higher in high-income countries among those with self-reported mental health problems, however, in most countries, it was less than 10%. This result is consistent with those of earlier surveys conducted in adult population in showing that the use of formal mental health care is lower in low-income countries [13]. Access to formal mental health services in low-income countries is limited, primarily due to resource constraints and challenges in service delivery including cultural stigma and lack of awareness [35]. The two exceptions were the two Nordic countries where almost one-third of girls with a high level of problems had sought professional help. The findings among the two Nordic countries could reflect their comprehensive and accessible mental health services, which include publicly funded healthcare systems, community-based care, a wide range of services from counselling to specialized psychiatric care, and a focus on mental health promotion and prevention [36]. For example, the ratio of child and adolescent psychiatrists per child is higher in European countries, particularly in Finland and Norway, compared to Asian countries [18]. Moreover, student welfare services are provided to all students in Finland and Norway by professionals with special competence in mental health issues including school nurses, psychologists and school counsellors, and there is strong emphasize on the wellbeing and mental health literacy in the school curriculum [21, 22]. However, surprisingly, in these Nordic countries with strong emphases on gender equality [37], the difference between girls and boys seeking formal help was strikingly high. In most countries, girls sought more help from professional sources than boys although the difference was not significant. Boys were less likely to perceive themselves as having difficulties, despite high scores in SDQ. In previous studies, boys have been found to seek less help for mental health problems [38], and this diminished help-seeking could be impacted by boys’ perceptions of their difficulties. This lower help-seeking behavior among boys could be attributed to the societal stigma leading boys to perceive seeking help as a sign of weakness, the gender stereotypes expecting reduced expression of emotions, and their preference for self-reliance [39].

Both specialized and primary health mental health services for adolescents are limited and almost nonexistent in many lower-income countries [29]. There is a lack of mental health workforce especially in lower-income countries possibly due to a lack of public funding [18]. The median spending on mental health per capita is merely 0.08 USD in low-income countries and 52.73 USD in high-income countries [29]. Large unmet need was observed in high-income countries. However, in the two Nordic countries, unmet need was significantly smaller among girls than in other countries. Adolescents commonly fail to seek help because of perceived stigma, embarrassment, and discrimination relating to mental illness [3, 5]. The main source of formal help was psychologists, counsellors and school nurses. Accessing help from a medical doctor was uncommon (0–20% among those who sought help).

Although this study shows a low rate of seeking formal help, many adolescents in all countries consider seeking help. A major challenge in adolescent mental health provision is how to lower the threshold for seeking help [40]. Efforts to address this challenge involve implementing interventions to reduce wait times for mental health services, providing low-threshold mental health services, and raising awareness to destigmatize seeking help [3]. In most countries, informal help-seeking was much more common than formal help-seeking and the difference was largest in lower-income countries. Especially in lower-income countries, relatives, peers and teachers are often the only help sources available for adolescents. In China and Vietnam, informal help-seeking was more than 14 times more common than formal while in India it was seven times more common. For example in Vietnam one in four girls considered but did not seek help, while more than 15% had sought help from informal sources and less than 1% had sought formal help. However, in three high-income countries (Finland, Israel and Norway), there was a reverse pattern, especially among girls, who had a two-to-three-fold increase in formal versus informal help-seeking.

An important finding was that informal sources of help are widely used among adolescents and are the key sources of help in many countries, especially in lower-income countries. Promoting help-seeking behavior and fostering inclusive spaces require collective action to go beyond the mere provision of professional care [41]. This could be achieved through targeted awareness campaigns and educational programs aimed at enhancing mental health literacy among adolescents, parents, teachers, and caregivers [42]. For example, interventions such as Transitions [43] and a mental health literacy intervention, MAKINGtheLINK [44] are shown to be effective in increasing help-seeking among adolescents. Additionally, digital mental health tools developed by professionals can be useful to motivate the help-seeking behavior among adolescents and to provide information about where and how they can seek help [45]. School-based mental health interventions are crucial as they offer the opportunity to provide support to a significant number of adolescents during their critical early stages of development [46]. The widely used informal sources of help especially in lower-income countries emphasize the importance of training non-professionals in providing mental health interventions [29].

### Limitations

The strengths of this study include the use of the same measures across countries, a large multinational sample of adolescents, and the investigation of not only help-seeking from formal help providers but also from informal sources. However, several limitations should be kept in mind when interpreting the results. First, although the study aimed to include both public and private schools in both urban and rural locations, the study was conducted in certain regions of the participating countries and the sample cannot claim to be fully representative of countries. Restricting the sample to adolescents currently present on the day of the survey may have led to some underreporting of mental health issues because of school absence. Second, the data are based on self-report and measurement error and recall and/or social desirability bias may be affecting the result. For example, although it was stated in the questionnaire that the focus of the survey was mental health of young people, the interpretation of terms like “problems, feelings, behavior or emotional trouble” in the help-seeking measure can be subjective. Having information from teachers and parents about adolescents’ mental health would have strengthened the study considerably. Third, important variables such as socioeconomic status, mental health literacy and previous experience with health services were not available in the survey dataset. The questionnaire did not include help-seeking behavior from social media which is now a source of help. Finally, the response options for gender were limited to “a boy” and “a girl”. To ensure inclusivity and representation in the data, it is essential for future research to include other options such as “transgender”, “non-binary/non-conforming” or “prefer not to respond”.

## Conclusion

The study findings demonstrate enormous global differences in mental health help-seeking behavior among 13 to 15-year-old adolescents representing economically very diverse societies. The findings emphasize the worldwide need to address adolescent mental health needs by a stepped-care model including community and family support, targeted psychological interventions delivered by trained non-specialist workers, and, finally, targeted intervention by specialists for those persons with the greatest needs. One of the most challenging barriers to service provision is the great shortage of skilled human resources to address adolescent mental problems, even in countries with public health care [47]. It is important to provide mental health literacy programs, community and school-based interventions and social-emotional learning programs to teach children that asking for help is a strength, not a weakness. New technological solutions using digitalized mental health interventions can help bridge the gap between limited resources and the growing demand for care while empowering individuals to take an active role in managing their mental well-being. However, digital interventions should be based on evidence of efficacy and be easily accessible.

## Electronic supplementary material

Below is the link to the electronic supplementary material.

Supplementary Material 1

## Data Availability

Raw data are not publicly available to preserve individuals’ privacy.

## References

[1] United Nations International Children’s Emergency Fund (2021) The State of the World’s Children On My Mind: Promoting, protecting and caring for children’s mental health. New York

[2] Solmi M, Radua J, Olivola M et al (2022) Age at onset of mental disorders worldwide: large-scale meta-analysis of 192 epidemiological studies. Mol Psychiatry 27:281–295. 10.1038/s41380-021-01161-7 34079068 10.1038/s41380-021-01161-7 PMC8960395

[3] Radez J, Reardon T, Creswell C et al (2021) Why do children and adolescents (not) seek and access professional help for their mental health problems? A systematic review of quantitative and qualitative studies. Eur Child Adolesc Psychiatry 30:183–211. 10.1007/S00787-019-01469-4 31965309 10.1007/s00787-019-01469-4 PMC7932953

[4] Finkelhor D, Turner H, LaSelva D (2021) Receipt of Behavioral Health Services among US Children and Youth with Adverse Childhood experiences or Mental Health symptoms. JAMA Netw Open 4:e211435–e211435. 10.1001/jamanetworkopen.2021.1435 33720370 10.1001/jamanetworkopen.2021.1435 PMC7961308

[5] Gulliver A, Griffiths KM, Christensen H (2010) Perceived barriers and facilitators to mental health help-seeking in young people: a systematic review. BMC Psychiatry 10:113–113. 10.1186/1471-244X-10-113 21192795 10.1186/1471-244X-10-113 PMC3022639

[6] Reynolds S, Wilson C, Austin J, Hooper L (2012) Effects of psychotherapy for anxiety in children and adolescents: a meta-analytic review. Clin Psychol Rev 32:251–262. 10.1016/j.cpr.2012.01.005 22459788 10.1016/j.cpr.2012.01.005

[7] Rickwood D, Thomas K (2012) Conceptual measurement framework for help-seeking for mental health problems. Psychol Res Behav Manag 5:173–183. 10.2147/PRBM.S38707 23248576 10.2147/PRBM.S38707 PMC3520462

[8] Zwaanswijk M, Van Der Ende J, Verhaak PFM et al (2005) Help-seeking for child psychopathology: pathways to Informal and Professional Services in the Netherlands. J Am Acad Child Adolesc Psychiatry 44:1292–1300. 10.1097/01.chi.0000181038.98712.c6 16292122 10.1097/01.chi.0000181038.98712.c6

[9] Angold A, Messer SC, Stangl D et al (1998) Perceived parental burden and service use for child and adolescent psychiatric disorders. Am J Public Health (1971) 88:75–80. 10.2105/AJPH.88.1.75 10.2105/ajph.88.1.75 PMC1508410 9584037

[10] Ghafari M, Nadi T, Bahadivand-Chegini S, Doosti-Irani A (2022) Global prevalence of unmet need for mental health care among adolescents: a systematic review and meta-analysis. Arch Psychiatr Nurs 36:1–6. 10.1016/j.apnu.2021.10.008 35094819 10.1016/j.apnu.2021.10.008

[11] Rens E, Dom G, Remmen R et al (2020) Unmet mental health needs in the general population: perspectives of Belgian health and social care professionals. Int J Equity Health 19:169. 10.21203/rs.3.rs-33443/v2 32993667 10.1186/s12939-020-01287-0 PMC7526210

[12] Alonso J, Codony M, Kovess V et al (2007) Population level of unmet need for mental healthcare in Europe. Br J Psychiatry 190:299–306. 10.1192/bjp.bp.106.022004 17401035 10.1192/bjp.bp.106.022004

[13] Demyttenaere K, Bruffaerts R, Posada-Villa J, in the World Health Organization World Mental Health Surveys (2004) Prevalence, severity, and Unmet need for treatment of Mental disorders. JAMA: J Am Med Association 291:2581–2590. 10.1001/jama.291.21.2581 10.1001/jama.291.21.2581 15173149

[14] United Nations Department of Economic and Social, Affairs PD (2019) World Population Prospects 2019. New York

[15] Quinlan-Davidson M, Roberts KJ, Devakumar D et al (2021) Evaluating quality in adolescent mental health services: a systematic review. BMJ Open 11:e044929. 10.1136/BMJOPEN-2020-044929 33972340 10.1136/bmjopen-2020-044929 PMC8112446

[16] Lynch L, Moorhead A, Long M, Hawthorne-Steele I (2023) The role of Informal sources of help in Young people’s Access to, Engagement with, and maintenance in Professional Mental Health Care—A Scoping Review. J Child Fam Stud 32:3350–3365. 10.1007/s10826-022-02498-5

[17] Petersen SZ (2020) Perfectionism’s relationship with higher education students’. A Literature Review, Help-Seeking

[18] Sourander A, Koskelainen M, Niemelä S et al (2012) Changes in adolescents mental health and use of alcohol and tobacco: a 10-year time-trend study of Finnish adolescents. Eur Child Adolesc Psychiatry 21:665–671. 10.1007/s00787-012-0303-8 22782292 10.1007/s00787-012-0303-8

[19] Sourander A, Chudal R, Skokauskas N et al (2018) Unmet needs of child and adolescent psychiatrists among Asian and European countries: does the Human Development Index (HDI) count? Eur Child Adolesc Psychiatry 27:5–8. 10.1007/s00787-017-1095-7 29288333 10.1007/s00787-017-1095-7

[20] Mori Y, Tiiri E, Lempinen L et al (2022) Feeling unsafe at School among adolescents in 13 Asian and European countries: occurrence and Associated factors. Front Psychiatry 13. 10.3389/fpsyt.2022.823609 10.3389/fpsyt.2022.823609 PMC9082541 35546950

[21] Department for General Assembly and Conference Management Regional groups of Member States In: United Nations. https://www.un.org/dgacm/en. Accessed 24 Jan 2023

[22] Sommar M (2016) Mental health among youth in Norway. Who is responsible? What is being done?

[23] Kivimäki H, Saaristo V, Wiss K et al (2019) Access to a school health nurse and adolescent health needs in the universal school health service in Finland. Scand J Caring Sci 33:165–175. 10.1111/scs.12617 30276842 10.1111/scs.12617 PMC7432168

[24] Akihiro N (2022) An overview of Non-school Mental-Health Support systems for children in Japan. Journal of Medical and Health Sciences

[25] Abram Sterne B Porter (2013) Overview of child and adolescent. Mental Health Services in Israel

[26] Peggy Karagianni (2016) Youth mental health context in Greece. Eur Health Psychol 18:119–122

[27] UNICEF Viet Nam (2018) Mental health and psychosocial wellbeing of children and young people in selected provinces and cities in Viet Nam

[28] Mehra D, Lakiang T, Kathuria N et al (2022) Mental Health interventions among adolescents in India: a scoping review. Healthc (Basel) 10:337. 10.3390/healthcare10020337 10.3390/healthcare10020337 PMC8871588 35206951

[29] Wang C, Zhang P, Zhang N (2020) Adolescent mental health in China requires more attention. Lancet Public Health 5:e637–e637. 10.1016/S2468-2667(20)30094-3 33271076 10.1016/S2468-2667(20)30094-3

[30] World Health Organization (2021) Mental Health ATLAS 2020. Geneva

[31] Sourander A, Koskelainen M, Helenius H (1999) Mood, Latitude, and seasonality among adolescents. J Am Acad Child Adolesc Psychiatry 38:1271–1276. 10.1097/00004583-199910000-00016 10517060 10.1097/00004583-199910000-00016

[32] Goodman R (1997) The strengths and difficulties Questionnaire: A Research note. J Child Psychol Psychiatry 38:581–586. 10.1111/j.1469-7610.1997.tb01545.x 9255702 10.1111/j.1469-7610.1997.tb01545.x

[33] Koskelainen M, Sourander A, Kaljonen A (2000) The strengths and difficulties Questionnaire among Finnish school-aged children and adolescents. Eur Child Adolesc Psychiatry 9:277–284. 10.1007/s007870070031 11202103 10.1007/s007870070031

[34] Goodman R (1999) The Extended Version of the strengths and difficulties Questionnaire as a guide to Child Psychiatric Caseness and Consequent Burden. J Child Psychol Psychiatry 40:791–799. 10.1111/1469-7610.00494 10433412

[35] Ojagbemi A, Gureje O (2022) Mental health in low- and middle-income countries. Oxf Textbook Social Psychiatry 699–712. 10.1093/MED/9780198861478.003.0072

[36] World Health Organization (2018) Mental Health ATLAS 2017. Geneva

[37] OECD (2018) Is the last Mile the Longest? Economic gains from gender Equality in Nordic Countries. OECD, Paris

[38] Häggström Westberg K, Nyholm M, Nygren JM, Svedberg P (2022) Mental Health problems among Young People—A scoping review of help-seeking. Int J Environ Res Public Health 19. 10.3390/ijerph19031430 10.3390/ijerph19031430 PMC8835517 35162452

[39] Bosco N, Giaccherini S, Meringolo P (2020) A gender perspective about young people’s seeking help. J Prev Interv Community 48:132–146. 10.1080/10852352.2019.1624353 31215327 10.1080/10852352.2019.1624353

[40] Kendrick T, Pilling S (2012) Common mental health disorders–identification and pathways to care: NICE clinical guideline. Br J Gen Pract 62:47–49. 10.3399/bjgp12X616481 22520681 10.3399/bjgp12X616481 PMC3252532

[41] Thornicroft G (2006) Shunned: discrimination against people with mental illness / Graham Thornicroft. Oxford University Press, Oxford

[42] Henderson C, Evans-Lacko S, Thornicroft G (2013) Mental illness stigma, help seeking, and public health programs. Am J Public Health (1971) 103:777–780. 10.2105/AJPH.2012.301056 10.2105/AJPH.2012.301056 PMC3698814 23488489

[43] Wei Y, Kutcher S, Austen E et al (2022) The Impact of Transitions, a Mental Health Literacy Intervention with Embedded Life Skills for Postsecondary students: preliminary findings from a naturalistic cohort study. Can J Psychiatry 67:452–461. 10.1177/07067437211037131 34379024 10.1177/07067437211037131 PMC9152239

[44] Lubman DI, Cheetham A, Sandral E et al (2020) Twelve-month outcomes of MAKINGtheLINK: a cluster randomized controlled trial of a school-based program to facilitate help-seeking for substance use and mental health problems. EClinicalMedicine 18:100225–100225. 10.1016/j.eclinm.2019.11.018 31922118 10.1016/j.eclinm.2019.11.018 PMC6948229

[45] Almeida R (2024) Beyond Textbooks and Standard Practices: Advancing Mental Health Literacy With Digital Tools. In: Emerging Technologies for Health Literacy and Medical Practice. pp 20–46

[46] Kutcher S, Wei Y, Costa S et al (2016) Enhancing mental health literacy in young people. Eur Child Adolesc Psychiatry 25:567–569. 10.1007/s00787-016-0867-9 27236662 10.1007/s00787-016-0867-9

[47] Patel V, Kieling C, Maulik PK, Divan G (2013) Improving access to care for children with mental disorders: a global perspective. Arch Dis Child 98:323–327. 10.1136/archdischild-2012-302079 23476001 10.1136/archdischild-2012-302079 PMC3672840

