# Supplementary material for: Unmet need for mental health care among adolescents in Asia and Europe
Source: Eur Child Adolesc Psychiatry. 2024 May 31;33(12):4349–59. doi: 10.1007/s00787-024-02472-0 (PMC11618194; doi:10.1007/s00787-024-02472-0)
Supplement: Supplementary file 1 — Supplementary Material 1 [file 787_2024_2472_MOESM1_ESM.docx]

Title: Unmet need for mental health care among adolescents in Asia and Europe

*European Child & Adolescent Psychiatry*

**Author names and affiliations**: Yuko Mori^1,2^, Andre Sourander^1,2,3^, Kaisa Mishina^1,2^, Tiia Ståhlberg^1,2,3^, Anat Brunstein Klomek^4^, Gerasimos Kolaitis^5^, Hitoshi Kaneko^6^, Liping Li^7^, Mai Nguyen Huong^8^, Samir Kumar Praharaj^9,10^, Henriette Kyrrestad^11^, Lotta Lempinen^1,2^, Emmi Heinonen^1^, the EACMHS Study Group

^1^Research Centre for Child Psychiatry, Department of Child Psychiatry, University of Turku, Turku, Finland

^2^INVEST Research Flagship Center, University of Turku, Turku, Finland

^3^Department of Child Psychiatry, Turku University Hospital, Turku, Finland

^4^Baruch Ivcher School of Psychology, Reichman University, Herzliya, Israel

^5^Department of Child Psychiatry, School of Medicine, National and Kapodistrian University of Athens, Aghia Sophia Children's Hospital, Athens, Greece

^6^Psychological Support and Research Center for Human Development, Nagoya University, Nagoya, Japan

^7^School of Public Health, Shantou University Medical College, Shantou, China

^8^Department of Psychiatry, Vietnam National Children’s Hospital, Hanoi, Vietnam

^9^Department of Psychiatry, Kasturba Medical College, Manipal, India

^10^Manipal Academy of Higher Education, Manipal, India

^11^Regional Centre for Child and Youth Mental Health and Child Welfare, Faculty of Health Sciences, UiT The Arctic University of Norway, Tromsø, Norway

Correspondence to Andre Sourander, MD, PhD, Research Centre for Child Psychiatry, Department of Child Psychiatry, University of Turku and Turku University Hospital, Lemminkäisenkatu 3/Teutori (3rd floor), 20014 Turku, Finland; e-mail: [andsou@utu.fi](mailto:andsou@utu.fi), +358 50 3653447

Table S1 Internal consistency of SDQ total difficulties score across countries

| Country |  | Cronbach’s Alpha |
| --- | --- | --- |
| Finland | Girls | 0.80 |
|  | Boys | 0.76 |
| Norway | Girls | 0.81 |
|  | Boys | 0.78 |
| Greece | Girls | 0.78 |
|  | Boys | 0.76 |
| Israel | Girls | 0.71 |
|  | Boys | 0.73 |
| Japan | Girls | 0.75 |
|  | Boys | 0.74 |
| India | Girls | 0.71 |
|  | Boys | 0.65 |
| Vietnam | Girls | 0.69 |
|  | Boys | 0.74 |
| China | Girls | 0.67 |
|  | Boys | 0.68 |
| Total | | 0.75 |

SDQ: The Strengths and Difficulties Questionnaire.

Table S2 Summary of the outcome and explanatory variables

| Variables |  | *n* | % |
| --- | --- | --- | --- |
| Age | 13 | 4303 | 32.6 |
|  | 14 | 5997 | 45.5 |
|  | 15 | 2884 | 21.9 |
| Gender | Girl | 6721 | 51.0 |
|  | Boy | 6463 | 49.0 |
| Help-seeking | No perceived need | 9390 | 71.2 |
|  | Considered getting help | 2526 | 19.2 |
|  | Sought informal help | 828 | 6.3 |
|  | Sought formal help | 440 | 3.3 |
| Emotional and behavioural difficulties (SDQ) | Total difficulties score >=  90^th^ percentile | 1458 | 11.1 |
| Perceived difficulties | No | 5049 | 49.0 |
|  | Mild | 4157 | 40.3 |
|  | Moderate/severe | 1099 | 10.7 |

SDQ: The Strengths and Difficulties Questionnaire.

**Table S3** The use of professional help among adolescents scoring above 90^th^ percentile total difficulties scores for total sample

| Country | Total  *n* | Above 90^th^ percentile SDQ total scores  *n* (*%)* | Formal help/adolescents with above 90^th^ percentile SDQ total scores^a^  *n* (*%)* | OR for unmet need  (95% CI) |
| --- | --- | --- | --- | --- |
| Finland | 2941 | 354 (12.0) | 90 (25.4) | 1 |
| Norway | 1831 | 218 (11.9) | 45 (20.6) | 1.28 (0.85 **–** 1.95) |
| Greece | 1024 | 113 (11.0) | 8 (7.1) | 4.42*** (2.03 – 9.61) |
| Israel | 1000 | 104 (10.4) | 7 (6.7) | 4.67*** (2.07 – 10.54) |
| Japan | 1783 | 200 (11.2) | 11 (5.5) | 6.00*** (3.01 – 11.97) |
| India | 1572 | 169 (10.8) | 4 (2.4) | 13.92*** (4.94 – 39.26) |
| Vietnam | 920 | 93 (10.1) | 1 (1.1) | 30.77*** (4.13 – 229.33) |
| China | 2039 | 207 (10.2) | 2 (1.0) | 34.08*** (8.15 – 142.57) |
| Total | 13110 | 1458 (11.1) | 168 (11.5) |  |

SDQ: Strength and Difficulties Questionnaire. OR: odds ratio. Bold type indicates statistical significance of at least p < 0.05. ^a^ The percentage of those who received professional help out of those who were above 90^th^ percentile of total difficulties score.

Table S4 Perceived difficulties among those with above 90^th^ percentile SDQ total scores

| Country | Total  *n* | No  *n* (*%)* | Mild  *n* (*%)* | Definite/severe  *n* (*%)* |
| --- | --- | --- | --- | --- |
| Girls | | | | |
| Finland | 242 | 21 (8.7) | 108 (44.6) | 113 (46.7) |
| Norway | 140 | 11 (7.9) | 18 (12.9) | 111 (79.3) |
| Greece | 68 | 5 (7.4) | 33 (48.5) | 30 (44.1) |
| India | 86 | 25 (29.1) | 31 (36.1) | 30 (34.9) |
| Vietnam | 46 | 3 (6.5) | 35 (76.1) | 8 (17.4) |
| China | 112 | 11 (9.8) | 68 (60.7) | 33 (29.5) |
| Total | 694 | 76 (11.0) | 293 (42.2) | 325 (46.8) |
| Boys | | | | |
| Finland | 110 | 28 (25.5) | 50 (45.5) | 32 (29.1) |
| Norway | 78 | 21 (26.9) | 20 (25.6) | 37 (47.4) |
| Greece | 44 | 6 (13.6) | 17 (38.6) | 21 (47.7) |
| India | 76 | 26 (34.2) | 28 (36.8) | 22 (29.0) |
| Vietnam | 47 | 5 (10.6) | 33 (70.2) | 9 (19.2) |
| China | 90 | 17 (18.9) | 47 (52.2) | 26 (28.9) |
| Total | 445 | 103 (23.2) | 195 (43.8) | 147 (33.0) |

SDQ: The Strengths and Difficulties Questionnaire.
